# Establishing Pragmatic Analytical Performance Specifications for Blood Beta-Hydroxybutyrate Testing

**DOI:** 10.1093/clinchem/hvad020

**Published:** 2023-03-15

**Authors:** Eric S Kilpatrick, Alexandra E Butler, Stephen L Atkin, David B Sacks

**Affiliations:** Department of Clinical Biochemistry, Manchester Royal Infirmary, Manchester, United Kingdom; Department of Postgraduate Studies and Research, Royal College of Surgeons in Ireland, Busaiteen, Bahrain; Department of Postgraduate Studies and Research, Royal College of Surgeons in Ireland, Busaiteen, Bahrain; Department of Laboratory Medicine, National Institute of Health, Bethesda, MD, United States

## Abstract

**Background:**

Currently, no authoritative guidelines exist recommending the analytical performance specification (APS) of blood beta-hydroxybutyrate (BOHB) testing in order to meet the clinical needs of patients. This study has applied existing diabetic ketoacidosis (DKA) BOHB diagnostic thresholds and the recommended rates of fall in BOHB concentrations during DKA treatment to establish pragmatic APSs for BOHB testing.

**Methods:**

Required analytical performance was based on 2 clinical requirements: (*a*) to reliably distinguish between non-adjacent DKA BOHB diagnostic categories of <0.6, 0.6 to 1.5, 1.6 to 2.9, and ≥3 mmol/L, and (*b*) to be assured that a measured 0.5 mmol/L reduction in BOHB indicates the true concentration is at least falling (meaning >0 mmol/L decline).

**Results:**

An analytical coefficient of variation (CV) of <21.5% could reliably distinguish all non-adjacent diagnostic categories with >99% certainty, assuming zero bias. In contrast, within-day CVs of 4.9%, 7.0%, and 9.1% at 3 mmol/L BOHB were required to assure truly falling ketone concentrations with 99% (optimal), 95% (desirable), and 90% (minimal) probability, respectively. These CVs are larger at lower BOHB concentrations and smaller at higher concentrations.

**Conclusions:**

Reliable tracking of changes in BOHB during DKA treatment largely drives the requirement for analytical performance. These data can be used to guide minimal, desirable, and optimal performance targets for BOHB meters and laboratory assays.

## Introduction

The use of blood ketone testing to diagnose and treat diabetic ketoacidosis (DKA) has largely replaced testing for urine ketones in guidelines and in clinical practice (1). The most commonly recommended test is beta-hydroxybutyrate (BOHB) which is the predominant ketone formed during DKA. Diagnostic BOHB thresholds now exist to define patients who have either developed DKA or are at a high risk of doing so (2, 3). If a diagnosis is made, there now exist recommended rates of fall in BOHB concentrations to help guide the success, or otherwise, of DKA treatment (4, 5).

Currently, there are no authoritative guidelines recommending analytical performance specifications of blood BOHB testing in order for it to meet the clinical needs of patients. This applies to both laboratory and point-of-care (POC) testing. Thus, we currently cannot be sure that the testing systems in use are performing sufficiently well in respect to bias and/or imprecision to be able to reliably diagnose or track the fall of BOHB concentrations in DKA patients. Lack of such guidance needs to be addressed, especially considering the increased reliance which is now being placed on these measurements.

Analytical performance specifications (APS) are used to describe the minimum, desirable, and optimal bias and/or imprecision when a laboratory test is used in patients within a specific clinical context (6). To derive these APSs, there is a hierarchy of evidence that is recommended to best inform what performance should be aimed for when using a particular test. The pinnacle of this hierarchy, as agreed at the 1st European Federation of Clinical Chemistry and Laboratory Medicine (EFLM) Strategic Conference in Milan (6), is to use data from clinical outcome studies to advise how well a test should perform to be clinically “fit for purpose” or better. As well as using direct outcome studies for this purpose (“Model 1a”), the APS for a test can be established indirectly (“Model 1b”) by using modelling or simulation to determine how analytical performance will impact the clinical classification of a patient or the medical decisions made for them. However, studies such as these do not exist for many assays and so, in their absence, a model based on the biological variation of a test is then recommended. Failing that being available, or if data on the biological variation of healthy individuals are known not to be applicable to those that are unwell, then basing assay performance on the “state-of-the-art,” i.e., the highest level of analytical performance achievable—at least by a percentage of laboratories or instruments—is advocated for use.

With regard to blood BOHB measurement, the biological variation in healthy individuals is unlikely to be appropriate for patients with suspected or confirmed DKA and, as stated above, it is not known whether state-of-the art assays perform adequately in clinical practice or not. In this study we therefore applied Model 1b principles by using both the existing DKA BOHB diagnostic thresholds and the recommended rates of fall in BOHB concentrations during DKA treatment to establish pragmatic APSs for blood BOHB testing.

## Materials and Methods

Table 1 describes the emerging consensus on thresholds used to categorize single concentrations of blood BOHB when diagnosing DKA (2, 3). Also included is the minimum rate of fall in BOHB recommended as part of the assessment of patients being treated for the condition (4,5). Based on these, the following clinical requirements were proposed by the authors to form the basis for establishing pragmatic BOHB analytical performance specifications.

**Table 1.** Diagnostic and treatment target thresholds for beta-hydroxybutyrate.

a. Diagnosis of diabetic ketoacidosis		
BOHB concentration	Description (Danne et al. (2))	Description (NG18 (3))
<0.6 mmol/L	Normal	Normal
0.6 to 1.5 mmol/L	Slightly increased risk of DKA	Ketonemia
1.6 to 2.9 mmol/L	Increased risk of DKA	Impending DKA
≥3 mmol/L^a^	Very high risk of DKA	Probable DKA

b. Treatment of diabetic ketoacidosis	
Recommendation	Testing frequency
>0.5 mmol/L/h fall in BOHB (Joint British Diabetes Societies for Inpatient Care (4))	Hourly
Approximately 0.5 mmol/L/h fall in BOHB (Wolfsdorf et al. (5))	Every 2 hours

Guidelines vary, with some defining this category as ≥3 mmol/L while other use >3 mmol/L.

1. When using the test to help diagnose DKA, it should have the ability to clearly distinguish between two non-adjacent BOHB group categories, i.e., it should be able to distinguish between a “normal” BOHB (<0.6 mmol/L) and “impending ketoacidosis” (1.6 to 2.9 mmol/L) and vice versa or tell apart a value in the 0.6 to 1.5 mmol/L category from one which is ≥3 mmol/L and vice versa. The corollary of being able to distinguish between non-adjacent categories is that a patient will not be incorrectly classified by more than one category. Not being able to make this distinction could lead to complete mismanagement of a patient.

2. When using the BOHB test to guide success of DKA treatment—where the target is for consecutive results to show a fall in BOHB of ≥0.5 mmol/L/hr—then, assuming that testing will initially be every hour, a fall of 0.5 mmol/L represents a high probability that the BOHB concentration is at least falling, i.e., decreasing by >0 mmol/L. For it truly to be rising in this situation would be an error which could directly impact on how a patient is treated.

We arbitrarily defined minimal, desirable, and optimal performance to indicate a 90%, 95%, and 99% probability of either (*a*) ensuring a single result used for DKA diagnosis was not actually beyond the intended or immediately adjacent diagnostic category, or (*b*) that an indicated fall of BOHB in 2 consecutive samples of 0.5 mmol/L was truly indicative of a reduction (>0 mmol/L) rather than an increase.

### Statistical Analysis

#### Single measurement for DKA diagnosis.

One-tailed *z* scores of 1.281, 1.644, and 2.326 were used to establish the total assay imprecision (standard deviation [SD] and coefficient of variation [CV]) required to ensure the BOHB test can distinguish between non-adjacent BOHB categories with probabilities of 90%, 95%, and 99% respectively. This represents the ability to differentiate 0.5 mmol/L from 1.6 mmol/L and vice versa, and between 1.5 and 3 mmol/L and vice versa.

#### Consecutive measurements to guide treatment.

From a purely analytical perspective, the *z* score (and thus the probability) of 2 consecutive measurements (X_A_ and X_B_) being different from one another is as follows (7):

Z = (X_A_-X_B_)/√2 SD

Rearranging this equation, one-tailed *z* scores of 1.281, 1.644, and 2.326 were again used to establish the assay imprecision (SD) required so that an indicated fall in BOHB of 0.5 mmol/L between 2 consecutive readings was either 90%, 95%, or 99% likely to indicate a true decrease (i.e., >0 mmol/L fall) from the first test value. It was assumed that the bias for a BOHB analyzer or assay would not differ between measurements. These exact calculations were verified as providing accurate probabilities by using a model involving 10 000 simulated pairs of measurements with 0.5 mmol/L difference and measurement SDs as derived from the above formula.

Rounding BOHB results to one decimal place introduces its own uncertainty, especially when looking for small changes between 2 consecutive measurements, For example, this factor alone means a 0.5 mmol/L difference can be reported when the true difference is anywhere between 0.40 and 0.60 mmol/L. Differences between these 2 extremes follow a triangular distribution whose standard uncertainty (approximating the SD) is defined as half the range of possible rounding differences between 2 consecutive tests divided by √6 (8). The square of this SD (the variance) was subsequently subtracted from the overall assay imprecision variances (calculated as above) to determine the remaining acceptable analytical variance.

## Results

### Single Measurement for DKA Diagnosis

**Fig. 1.**
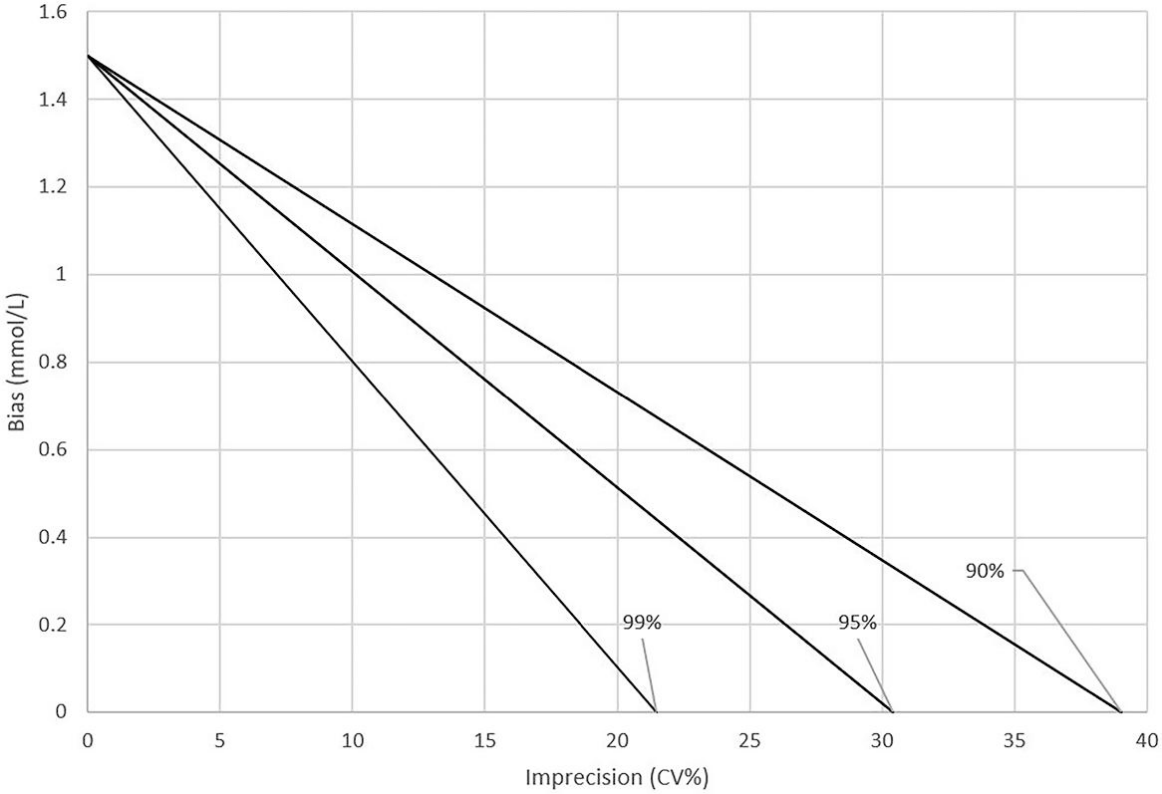
Effect of analytical bias on required assay imprecision at a beta-hydroxybutyrate concentration of 3 mmol/L with 99%, 95%, and 90% probability of not miscataloging the result by more than one category.

Table 2 describes the analytical total SDs and CVs required to reliably distinguish between non-adjacent diagnostic categories with 90%, 95%, and 99% probability, assuming no assay bias. The lowest CVs related to imprecision at 3 mmol/L BOHB. Figure 1 shows how these CVs at 3 mmol/L fall with increasing assay bias.

**Table 2.** Assay SD and CV targets to distinguish between categorical beta-hydroxybutyrate concentrations with varying certainty, assuming no analytical bias.^a^

	Distinguish 0.5 from 1.6 mmol/L BOHB and vice versa			Distinguish 1.5 from 3.0 mmol/L BOHB and vice versa		
Probability	SD, mmol/L	CV at 0.5 mmol/L, %	CV at 1.6 mmol/L, %	SD, mmol/L	CV at 1.5 mmol/L, %	CV at 3.0 mmol/L, %
90%	0.858	172	53.6	1.17	78.0	39.0
95%	0.669	134	41.8	0.912	60.8	30.4
99%	0.473	95	29.6	0.645	43.0	21.5

SD and CV representative of combined between- and within day imprecision.

### Consecutive Measurements to Guide Treatment

#### Total analytical imprecision.

Table 3 shows the total assay imprecision limits (expressed as SDs) which will assure that a 0.5 mmol/L reduction between consecutive measurements represents a true fall in BOHB with 90, 95, or 99% certainty. These were validated by the simulated data as being accurate.

**Table 3.** Assay imprecision limits (SD) where a measured 0.5 mmol/L fall assures a reduction between consecutive beta-hydroxybutyrate measurements with 90%, 95%, or 99% certainty, assuming constant analytical bias.

	BOHB imprecision	
Probability	Total assay SD, mmol/L	Analytical SD, mmol/L^a^
90%	0.276	0.273
95%	0.215	0.211
99%	0.152	0.146

Accounting for rounding errors. Representative of within-day SD.

#### Rounding error imprecision.

As described in the Materials and Methods, the influence alone of rounding BOHB values to one decimal place is such that a measured fall of 0.5 mmol/L between 2 consecutive measurements can be due to a true concentration change of 0.5 ± 0.1 mmol/L. The standard uncertainty (representing the SD) of this distribution is therefore 0.1/√6, or 0.041 mmol/L. Table 3 shows the slightly tighter analytical imprecision targets required as a consequence of rounding contributing imprecision of its own.

The effect of rounding errors on diagnosing patients with a single measurement was found to be negligible and so was not applied to this situation.

#### Coefficient of variation targets.

The 0.5 mmol/L/hr recommended rate of BOHB reduction applies to any initial BOHB value, so while the SD targets are therefore the same throughout an assay’s measurement range, target CVs vary according to the examples given in Table 4.

**Table 4.** Assay imprecision limits (CV) where a measured 0.5 mmol/L fall assures a reduction at selected initial beta-hydroxybutyrate concentrations, assuming constant analytical bias.

	Initial BOHB concentration					
	1.0 mmol/L	1.5 mmol/L	2.0 mmol/L	2.5 mmol/L	3.0 mmol/L	6.0 mmol/L
**Probability**	**Target CV (%)^a^**					
90%	27.3	18.2	13.6	10.9	9.1	4.5
95%	21.1	14.1	10.5	8.4	7.0	3.5
99%	14.6	9.8	7.3	5.9	4.9	2.4

^a^Representative of within-day CV.

## Discussion

Blood BOHB measurement, especially at POC, has supplanted urine ketone testing in most contemporary diabetes guidelines as a means of identifying patients with developing or overt ketoacidosis (2, 3). It is also now recommended as an important tool when assessing the success of DKA treatment (4, 5). This study has used these diagnosis and treatment guidelines to help determine how well a BOHB assay should perform so that the results can be relied upon to guide patient management.

This “indirect outcome study” approach would have merit even if there were alternatives, but it is of particular value because other means of deriving assay performance specifications do not apply well to BOHB measurement. The reasons for this are: firstly, there is a paucity of direct outcome data related to ketone measurement; secondly it is doubtful the biological variability of healthy subjects can be applied to DKA patients; and lastly, it is not known if the current state-of-the-art performance of assays meet clinical requirements.

The rationale for wishing to be able to consistently discriminate between 2 non-adjacent diagnosis categories arises partly from the difficulty of any test dependably classifying disease or health when using a single cutoff. It is therefore helpful that there are 4 clinical categories commonly in use (Table 1) which form a gradient of severity and intervention where being incorrectly classified by no more than one category is still likely to mean most patients are given further attention when required.

In fact, the BOHB analytical performance required to achieve this aim does not immediately appear onerous, with a CV of better than 21.5% assuring that this will be the case with at least 99% probability at all relevant BOHB concentrations (Table 2). However, this imprecision refers to all sources of random error combined (including within- and between-day variation) and does not take account of systematic bias that could exist due to, for instance, changes in reagent/strip lots. Figure 1 shows how these allowable CVs can fall with increasing assay bias. Thus, for example at 3 mmol/L, while a CV of 21.5% allows there to be at least 99% certainty that patients are not completely miscategorized when there is no assay bias, a 0.8 mmol/L bias means the CV can only be 10% in order to be equally assured.

Confidently tracking falls in BOHB in patients being treated for DKA fundamentally appears more challenging. This is because 2 measurements are being made (which can compound analytical errors, including those associated with rounding to one decimal place) and the changes being sought are relatively small (0.5 mmol/L), irrespective of the first test concentration. Balancing this, assay bias is probably less of an issue if using the same meter or assay for both tests and, being measured so closely together, the random error is mainly reflective of only within-day imprecision.

Table 3 shows the analytical SDs required to ensure with either 90%, 95%, or 99% certainty that a 0.5 mmol/L BOHB fall does not, in fact, truly reflect a rise in ketone concentration. If an indicated satisfactory fall in BOHB were genuinely due to a rise, then this could be detrimental to a patient since their DKA treatment would be less likely to be intensified when it otherwise might need to be. Since the SDs are constant throughout the analytical measuring range, it means the target CVs necessarily vary according to the concentration measured, as shown in Table 4, with lower CVs being required as the BOHB concentration rises. All these analyses assume the same meter or assay will be used on the same patient and underlines the importance that different meters or assays not be used during the same DKA episode.

When comparing these proposed performance specifications for tracking with data from recent literature, a study of 4 BOHB meters from 2 manufacturers found within-run SDs to vary from 0.05 to 0.29 mmol/L at BOHB levels ranging from 0.16 to 4.40 mmol/L (9). Amongst the 3 meters where one of their QC SDs did not meet the 95% desirable probability target for assuring a fall in BOHB (an SD value of 0.21 mmol/L, Table 3), all were at the highest QC concentration tested (9). This observation—that the largest SDs were at the highest measured BOHB concentrations—was also found to be the case in other studies (10–12) and so there may be a particular issue in tracking BOHB in patients who have higher initial values, especially around the 6.0 mmol/L concentration indicative of “severe DKA” (4). Mitigating this risk is the fact that the treatment of these particularly unwell patients should not be solely guided by BOHB measurements but also include the findings from other biochemical assessments (such as pH and blood glucose) as well as from clinical observations.

Categorizing patients using the <0.6, 0.6 to 1.5, 1.6 to 2.9, and ≥3 mmol/L criteria introduces further potential errors, such as those due to between-run imprecision and bias. Existing meter evaluation studies suggest that combining between-run with within-run imprecision continued to meet the 99% criteria (Table 2) for discerning non-adjacent categories (data not shown) but only assuming there was no assay bias (9, 10, 12). Assay bias when compared to reference laboratory methods (either positive or negative, depending on the meter) was indeed also a feature of these studies, particularly at high BOHB levels, although it was less of an issue below 3 mmol/L where, as demonstrated in Fig. 1, this could have the greatest impact on patient categorization.

This analysis is not without its limitations. Firstly, it is based on existing clinical guidance which may be subject to change. The types of random and systematic errors included have also been simplified and, while they are likely to reflect the largest sources of uncertainty, they may not be entirely representative of all that could impact on a test result.

In conclusion, this study has used current clinical guidelines to derive pragmatic minimal, desirable, and optimal BOHB assay performance specifications. These were driven largely by the requirement to reliably track changes in BOHB during DKA treatment. They will hopefully help enlighten test users on how much reliance can be placed on BOHB measurement and also inform meter or assay manufacturers where their products could be further improved.

## Employment or Leadership

E.S. Kilpatrick, *Clinical Chemistry*, AACC; D.B. Sacks, *Clinical Chemistry*, AACC.

## Consultant or Advisory Role

None declared.

## Stock Ownership

None declared.

## Honoraria

None declared.

## Expert Testimony

None declared.

## Patents

None declared.
